# Cuffless Blood Pressure Measurement Using Linear and Nonlinear Optimized Feature Selection

**DOI:** 10.3390/diagnostics12020408

**Published:** 2022-02-05

**Authors:** Mohammad Mahbubur Rahman Khan Mamun, Ali T. Alouani

**Affiliations:** Department of Electrical and Computer Engineering, Tennessee Technological University, Cookeville, TN 38501, USA; aalouani@tntech.edu

**Keywords:** electrocardiogram, photoplethysmography, symmetric uncertainty, fast correlation, ReliefF algorithm, blood pressure measurement

## Abstract

The cuffless blood pressure (BP) measurement allows for frequent measurement without discomfort to the patient compared to the cuff inflation measurement. With the availability of a large dataset containing physiological waveforms, now it is possible to use them through different learning algorithms to produce a relationship with changes in BP. In this paper, a novel cuffless noninvasive blood pressure measurement technique has been proposed using optimized features from electrocardiogram and photoplethysmography based on multivariate symmetric uncertainty (MSU). The technique is an improvement over other contemporary methods due to the inclusion of feature optimization depending on both linear and nonlinear relationships with the change of blood pressure. MSU has been used as a selection criterion with algorithms such as the fast correlation and ReliefF algorithms followed by the penalty-based regression technique to make sure the features have maximum relevance as well as minimum redundancy. The result from the technique was compared with the performance of similar techniques using the MIMIC-II dataset. After training and testing, the root mean square error (RMSE) comes as 5.28 mmHg for systolic BP and 5.98 mmHg for diastolic BP. In addition, in terms of mean absolute error, the result improved to 4.27 mmHg for SBP and 5.01 for DBP compared to recent cuffless BP measurement techniques which have used substantially large datasets and feature optimization. According to the British Hypertension Society Standard (BHS), our proposed technique achieved at least grade B in all cumulative criteria for cuffless BP measurement.

## 1. Introduction

Cardiovascular diseases (CVD) are responsible for major health concerns around the world. Potential symptoms or information which may provide early prediction of CVD may help many people to take proper precautionary measures [1,2]. Consistent high blood pressure (BP) is one of the four major risk factors for CVD and the main reason for hypertension, so frequent monitoring and tracking of blood pressure trends should be prioritized [3]. Chronic hypertension may cause damage to several organs [4]. So, the early detection of hypertension allows patients to take treatment at an early stage to reduce the possibility of deteriorating cardiac condition due to hypertension which does not show symptoms at an early stage. The common BP measurement device cannot detect the “white coat hypertension” syndrome [5], on top of that the traditional device to measure BP uses techniques such as oscillometric [6] and auscultation [7], which are useful for infrequent measurements but not continuous. In addition, even when the patient tried to measure BP more frequently using these techniques, the amount of discomfort and inconvenience makes it unsuccessful [8]. The proper way to get a continuous cuffless blood pressure measurement with gold-standard accuracy is catheterization which requires a medical facility with professional intervention [9,10]. So, finding a solution for the frequent measurement of blood pressure during daily life in a cuffless and noninvasive manner remains a topic of research. The goal is to develop frequent BP measurement devices that are non-invasive, inexpensive, cuffless, wearable, and convenient.

Over the past few years pulse transit time, which is the time it takes for the pressure wave to go from one area of the body to another, has attracted a good amount of attention as a characteristic of blood flow pattern behavior in the artery to measure blood pressure [11,12,13,14,15,16,17]. Using photoplethysmography (PPG) and electrocardiography (ECG), the PAT can be measured. However, PEP can be influenced by different factors such as stress, age, emotion, movement, etc. In addition, it was found that PAT does not similarly correlate with DBP as with SBP [18,19]. Blood is pushed through the vessels when the heart ventricular chamber contracts maintaining expansion and contraction [20]. This regular activity impacts significantly the elastic nature of vessel walls and over time the elastic nature of vessel walls naturally degrades which in turn affects the pressure of the blood as well as velocity of pulse wave [21]. However, the accuracy is not consistent because, due to factors such as age, diet and stress, etc., the elasticity varies between individuals [22,23,24]. Along with PTT and PAT, the use of only a single sensor such as PPG also gained large research momentum recently. Due to being optical and very inexpensive, it became very popular among researchers to extract a different number of features from PPG waves and use formulas motivated by fluid dynamics or machine learning to come up with a model to measure blood pressure [25,26,27,28,29,30]. Volumetric change in the peripheral artery and blood pressure change is correlated [31]. This characteristic is also being used along with some other features from the PPG wave to make a model for BP measurements through different machine learning algorithms [32,33,34,35,36]. These techniques heavily depend on the accurate acquisition of PPG signals, on top of that there is yet to be a general formula or technique which works across different databases with equal accuracy.

Improvement in digital signal processing through the use of filters made it possible to easily process biomedical signals [37,38,39,40,41,42,43,44,45]. The motion artifacts, due to movement, and the heavy deviation of results, due to change in datasets, still pose a significant challenge [33] in adopting existing data-driven techniques. Evaluation with individual or another new dataset their performance fails to uphold with an acceptable accuracy range that is given by the Association for the Advancement of Medical Instrumentation (AAMI) [46]. In solving classification and pattern recognition deep learning is now a very effective and promising tool. Deep learning has the advantage of learning the features directly from the raw signals and being applied in different fields such as image recognition, speech recognition, and object identification, etc., [47,48,49,50]. A large amount of medical data is being mined for diagnostic values such as cancer detection, heart beat classification, heart disease detection brain tumor recognition, etc., [51,52,53]. Deep learning already started to play a vital role in research related to heath states, detecting diseases, performing diagnoses, and taking preventive measures. Liang et al. proposed blood pressure measurement technique using wavelet transformation and CNN from PPG signal. The accuracy found from that experiment was 82.9% [54]. Another study adopted a model that consisted of artificial neural network in lower level for temporal feature extraction and LSTM layers in upper layer for time domain variation in ANN layer features [55]. Unfortunately, the experiment with only 39 subjects showed greater accuracy but they have not replicated it over larger datasets. Shimazaki et al. proposed a different experiment [39] using a three-layer auto-encoder to generate the features automatically and estimate BP [56]. Along with PPG, speed of PPG, derivative of PPG and health data such as age, height, weight, sex, drug presence, and pulse rate were used as inputs. The resulted deviation gold standard was 11.86 which was still quite high. Eom et al. proposed CNN-based BP estimation. Due to a very small population number (only 15), the small standard deviation is not a significant improvement [57]. Athaya and Choi proposed [58] a PPG-based deep learning technique (U net) to measure blood pressure with a significantly small standard deviation, but the population was only 100. Aguiree et al. proposed RNN encoder-decoder architecture whose performance showed large estimation errors and standard deviation of 7.32 and 15.67 for DBP and SBP [59]. All of these aforementioned experiments were done during the last three years and considering their performance it is evident that the cases which achieved better accuracy or small standard deviation have smaller datasets or populations, and when the larger datasets or different individuals from the original dataset were used, they cannot sustain the same performance [60]. Recently, researchers used deep learning techniques to measure BP. The performance showed better accuracy in contemporary methods while using the same dataset for training and testing. Still, several shortcomings remain; first, the testing was done using the same dataset, so the higher accuracies do not carry over to new datasets or individuals. Second, in rare cases when they have chosen to test the technique with separate datasets, the size of those datasets was very small. Third, since deep learning takes care of feature extraction and selection without human intervention, it is always difficult to explain the relationship between any feature and outcome [57,61,62,63,64,65].

To perform feature selection and optimization, the most common and popular choice is the use of Pearson’s correlation coefficient. It results in a very accurate correlation whenever the features are linear or very close to being linearly related. However, when variables are nonlinearly related, Pearson’s correlation coefficient is calculated using linear approximation between such variables. Based on how nonlinear the relation is, the Pearson coefficient may or may not provide an accurate correlation value. If from beforehand it is known that the relation among some of the features is highly nonlinear, Pearson’s coefficient may provide the wrong feature selection [66]. This could impact the accuracy of the BP measurements. A potential solution would be to calculate the correlation to measure independence between features irrespective of their linear or nonlinear relationship. The relationship among physiological measurements, such as the heart’s electrical activity, pulse wave, and blood circulation through arteries, etc., are highly nonlinear [67,68,69]. For example, blood pressure and artery diameter or stiffness are related in a strong nonlinear manner and any sort of linear approximation would result in a much smaller correlation value compared to their existing nonlinear relation between feature and target variable [67]. The nonlinear dependencies decrease the chance of reducing the dataset dimensions since linear techniques or linear approximation may fail to sufficiently identify them [70]. In addition, extreme outliers or skewed distribution of data, which are common in physiological datasets, may negatively impact the performance of linear methods [70]. In contrast to the only linear model, the nonlinear models based on both linear and nonlinear dependencies show better feature selection performance with more stability and are less affected by including or excluding variables from the dataset [71,72]. For example, research was done by extending the linear correlation to an alpha-grade monomial relation who maximizes the correlation based on changing the value of alpha to which type of nonlinear relationship data exhibit [71]. Their study proved that the proposed alpha-grade correlation coefficient overcame the common drawback of statistical measure that evaluates the significance of relationship between two variables based on their linearity. These studies have provided the basis for the potential improvement of feature selection techniques for physiological data. The expectation is it will solve all aforementioned shortcomings by considering the correlation between both linearly and nonlinearly related features from vital signs, getting rid of bias towards high cardinality features and allowing total correlation in place of only pairwise.

Based on the literature survey it is evident that there is a need for a reliable and accurate cuffless blood pressure measurement system that can be used frequently by a person at his/her convenience. In this paper, a cuffless BP measurement technique that accounts for linearly and nonlinearly dependent feature with blood pressure is proposed. For the first time, a hybrid feature selection algorithm based on multivariable symmetric uncertainty (MSU) is used to optimize the number of relevant features and reduce the number of redundant and nonlinearly related to BP. The MSU-centered fast correlation and ReliefF feature selection techniques can identify dependence among features irrespective of whether they are linearly or nonlinearly related compared to the popular Pearson’s coefficient which only deals with linearly dependent features. The use of the mentioned hybrid feature selection algorithm is expected to give more accurate measurements than what is currently available in the state of the art. The rest of the paper is organized as follows: Section 2 focuses on the proposed technique for BP measurement using biomedical signal, Section 3 discusses data analysis followed by Section 4 with the result and performance of the technique using an online dataset and finally, Section 5 concludes the paper and discusses future work

## 2. Materials and Methods

**Figure 1:**
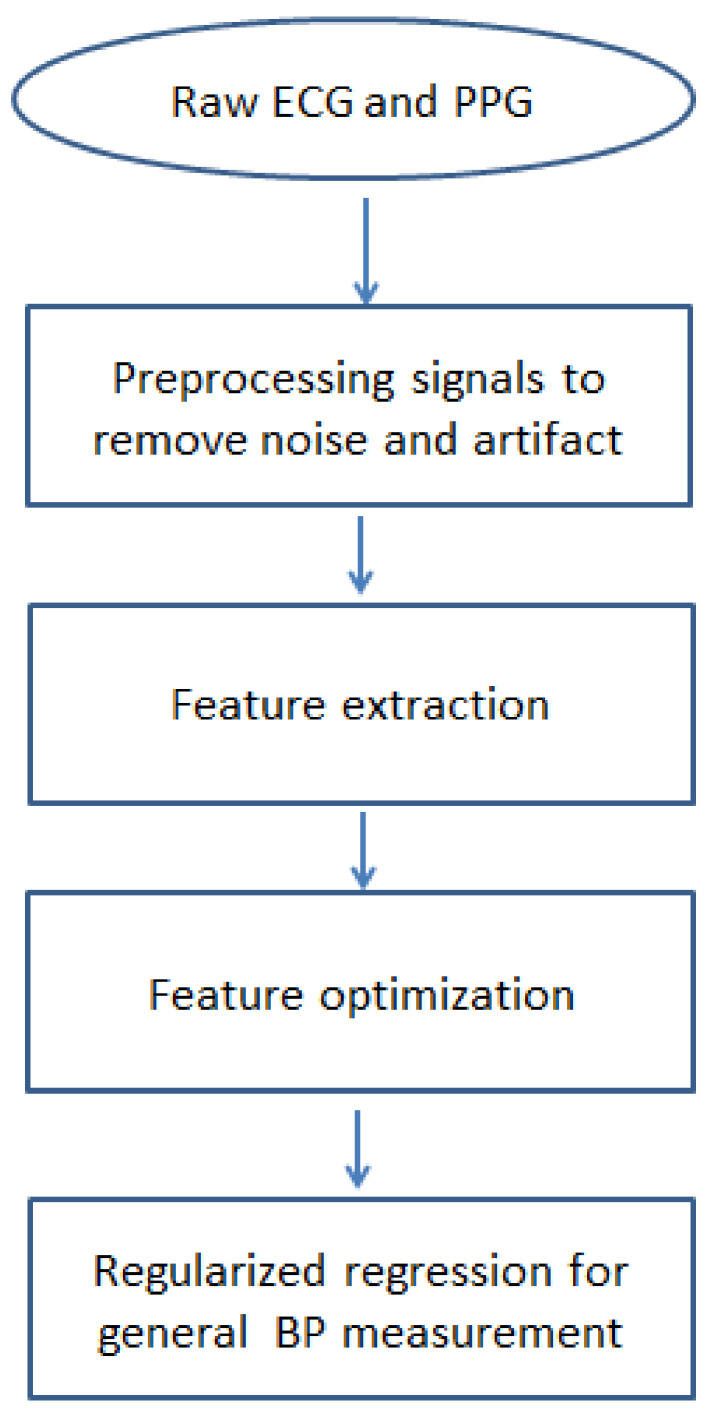
Diagram for model used for cuffless BP measurement.

The significant issues which were addressed in the technique are: first, to select the biomedical signals and features that have noticeable biological impacts on change in blood pressure; second, using symmetric uncertainty to perform a fusion of the fast correlation and ReliefF algorithms to optimize feature selection; third, to use regularized regression to develop a relationship between the extracted features and BP measurements. As the primary features selection is done considering their biological significance and the feature optimization algorithm is performed based on total correlation rather than just pairwise, this technique is expected to result in a smaller number of predominant features. The penalty-based regularization technique used in this study solves the problem of the unbalanced result of bias-variance found in ordinary least square regression. In Figure 1, a flowchart shows the steps of the proposed measurement technique. In subsequent subsections, these steps will be described in detail. 

When the raw signal is used for this kind of study the best way to proceed with that is to annotate them into acceptable and unacceptable signals. The recording of the measurement of vital signs can deviate from the ideal case due to so many reasons other than the physical deterioration of patients. So, without annotation, it is hard to differentiate between acceptable and unacceptable signals. In this study, annotation was not performed, and we tried to find the deviated signals by using manual inspection. Ideally using an index such as signal-to-noise ratio or some other statistical parameters can be used to use only acceptable signals based on a preferred threshold. A basic classifier can be used to find out which index would perform the best. Another limitation of this kind of study is the choice of location from where the vital signs were recorded or measured. Since the dataset used in our research was from the MIMIC-II dataset, which is data acquired from hospital settings from ICU patients, the physical locations of data acquisition from patients were predefined. Study [73] shows wrist PPG is difficult to measure accurately which results in an error in peak detection; compared to that, head and finger is the much better choice. Since the distance travel by the pulse originated at the heart plays a significant part in calculating blood pressure, the best case would be to use multiple places to measure PPG signals to validate each other’s data and make the measurement of pulse transit time more reliable.

Different lead information of ECG records the electrical activity of the heart from different directions and orientations. Ideally, all 12-lead information is the best way to select any feature necessary from ECG but to make the data acquisition inexpensive and the calculation simple it is hard to accommodate more than one or a few leads at the same time. It will become cumbersome for the patient to measure the signal as well; on the other hand, not having information from all the leads deprives them of having some necessary information or the ability to cross-check any specific deviation in ECG wave. This study put focus on making the system as simple as possible while keeping accuracy and feature number optimized, so only lead information from lead II was used. The use of more lead information may help to bring forth more features that are dependent on consecutive lead information. The intrinsic stiffness of the carotid artery was only shown to increase for young patients irrespective of the change in BP but for the elderly, it is the opposite [74]. In addition, a continuous increase in BP enhances increases in vascular thickness and structural stiffness, and load on the arterial wall [75]. Damage to either small or large arteries affects the rise of central BP by increasing more wave reflections during blood flow [76]. All this information points directly to the necessity of accurately measuring the change in arterial stiffness as it changes due to the physical changes of patients, so measuring the changes in arterial stiffness using a separate sensor would be ideal.

The inclusion of statistical and spectral features along with morphological features has both positive and negative aspects for measuring blood pressure. On the positive side, more features would increase the chance of getting more relevant features in the final feature list as well as have the advantage of observing the change in features from another perspective such as statistical or frequency wise. On the negative side, the use of statistical features carries the risk of misinformation due to the improper segmentation of signals; also, lack of information about how the frequency components are directly related to the change in blood flow pose a risk of either accepting a frequency that has a minimum effect or neglecting one with higher impact. Another common limitation of this kind of study is the lack of a centrally managed dataset to test the produced model. The best way to test a trained model is to train with one standard database and test with another standard database, but unfortunately, the unavailability of biomedical data collected reliably for a longer period with high-quality raw data is still difficult to find from several sources. These drawbacks create several bottlenecks: first, people are forced to acquire data by themselves and with a limited number of data it is quite impossible to bring forth any robust model; second, even if one’s model shows very high accuracy, it could fail to hold similar accuracy when another dataset is used to validate the performance. Since the datasets are differently standardized, it is very difficult to conclude whether the difference in performance came due to a difference in dataset or technique.

### 2.1. Selection of Vital Sign

The pressure on the walls due to blood flowing inside the vessels is proportional to the force that the heart uses to pump blood; this pressure is called blood pressure. The flow of blood is proportional to the pressure gradient which is the force that pushes the blood along the vessels and is inversely proportional to the resistance against the flow. From the aorta and throughout the cardiac cycle, the pressure is highest but when the large vein brings the blood back to the heart it becomes lowest and is around 0 in the vena cava area. These facts indicate that the pressure gradient is proportional to the blood pressure throughout the heart cycle [77].

**Figure 2:**
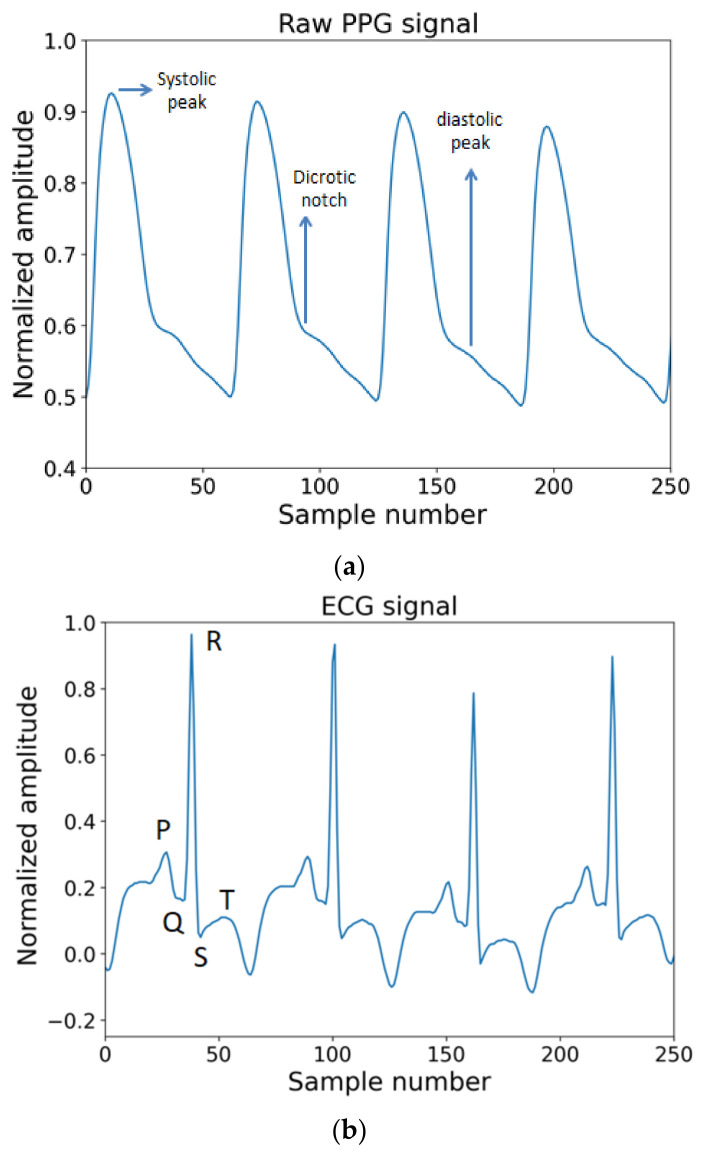
(**a**) A single PPG signal. (**b**) Electrical activity pattern from ECG wave.

The most commonly known reason behind hypertension is a build-up of plaque in arterial walls and a decrease in the elasticity as well as the radius of arteries requiring more force from the heart to pump blood and higher pressure on the vessel wall. This plaque build-up impacts patients’ health in two ways. First, it progressively worsens the health condition by obstructing blood flow to different important organs of the body and second, it brings significant cardiovascular disorders. This knowledge guided us to choose appropriate vital signs which reflect changes in blood pressure. As explained in what follows, the ECG and PPG waves are the proper biomedical signals to be used to perform cuffless BP measurements. Consider Figure 2a,b, where PPG and ECG waves are shown.

In the ECG wave, when the R peak in the QRS complex of the ECG wave occurs, that means the left ventricle forces the blood out to the aorta. Now when this rush of blood flow reaches the place where the PPG wave was measured, the time interval between the R peak and corresponding to any significant points in PPG wave, such as systolic peak, diastolic peak, point with the highest slope, dicrotic notch, or start point of systolic phase, etc., can be measured. These time intervals have a highly correlated relation with changes in blood pressure [78]. In a study using the Windkessel model simulation, a trend line was obtained by measuring systolic rise time (SRT) and diastolic fall time (DFT) for two different compliances where compliance is the ability of the vessel to extend or be elastic enough to respond to an increase in pressure due to a larger amount of blood [78]. Blood pressure is predominantly affected by the compliance of the vessel wall. The compliance works in inverse order compared to tension or stiffness in the artery walls. The behavior from simulation also resembles the behavior of vascular change such as an increase in compliance and decrease in DFT peak [78]. The reason is when artery blood pressure increases at the same time the stiffness or tension in vessel increases which in turn decreases the compliance. With the opposite change in blood pressure, the action reverses in terms of compliance too.

### 2.2. Pre-Processing Signals

**Figure 3:**
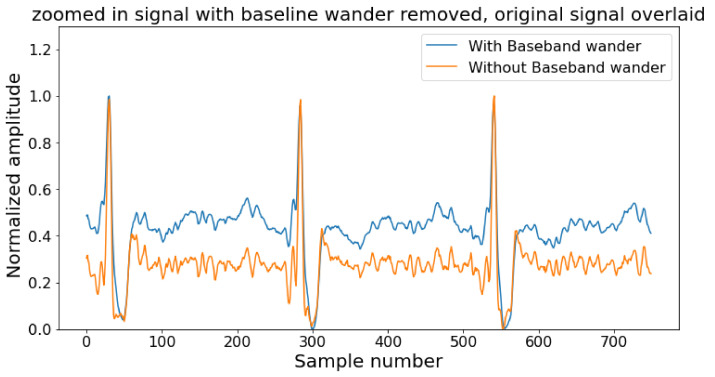
Example of normal noise-free and noisy signal.

Contamination comes in the ECG wave from noise and artifacts, and the goal of pre-processing step is to reduce them as much as possible keeping the integrity of the original signal [79]. The noise and artifacts are power line interference (50–60 Hz), base-band wanders (around 0.3 Hz), power line interference, motion artifacts, and noise from nearby electronic devices, etc. The pre-processing stage starts with a filtering block to delete the artifacts from the ECG signal [80]. An example of noisy and noise-free ECG signals is depicted in Figure 3. Typically, a band stop filter and an FIR filter of order 50 is sufficient to get rid of noise with cut-off frequencies of 0.5 Hz and 100 Hz [81]. The output of the FIR filter is being passed through a moving average filter to smooth the signal and remove the unusual momentary spike. Unlike the low pass filter, the high pass filter does not attenuate much of the signal but suffers from phase shifts [82]. The high pass filter removes the DC offset which comes from electrode/gel/body interference and is used to remove the baseband wanderer.

**Figure 4:**
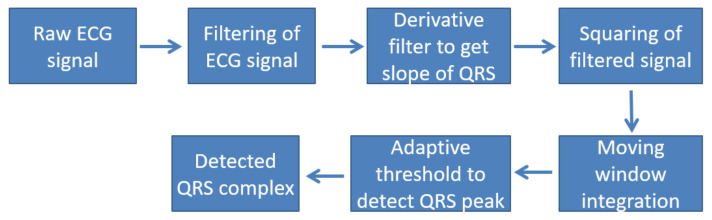
Block diagram of Pan–Tompkin’s algorithm [83].

Normalization is another step in the pre-processing stage which helps us to make comparisons among data from different patient at the same time. One of the most important pre-processing techniques is the use of the Pan and Tompkins algorithm [83] which includes the differentiation, squaring, and finding of the peak. In Figure 4, the flowchart for the Pan and Tompkins algorithm is depicted.

PPG and ABP waves have similarities in terms of morphological aspects at least; FFT was used to remove high frequency noise. In lower frequency regions, a segment from 0.15 to 0.4 Hz, which is a consequence of respiratory effect, and another segment from 0.01 to 0.15 Hz vascular resistance, are divided [84]. Although in numbers there is coherence between the lower frequency region of PPG and BP, the mechanism or rationale behind the coherence is still debated among researchers [84]. Change in respiration rhythm is reflected in the change in stroke volume which in turn affects PPG wave pattern. Although researchers are not yet agreed on how much respiration affects pulse wave, it is agreed that the change is very minor when only amplitude and slopes are in question. The ideal way is to reduce or nullify the impact of respiration on PPG wave pattern by leveling the PPG wave without the envelope created by respiration.

The main peak of each PPG cardiac cycle was detected through Daubechies 3 wavelet. The DB3 is similar to the changes in PPG wave. The common peaks and intervals in PPG waves are systolic peak, diastolic peak, systolic time, diastolic time, and dicrotic notch. 

**Figure 5:**
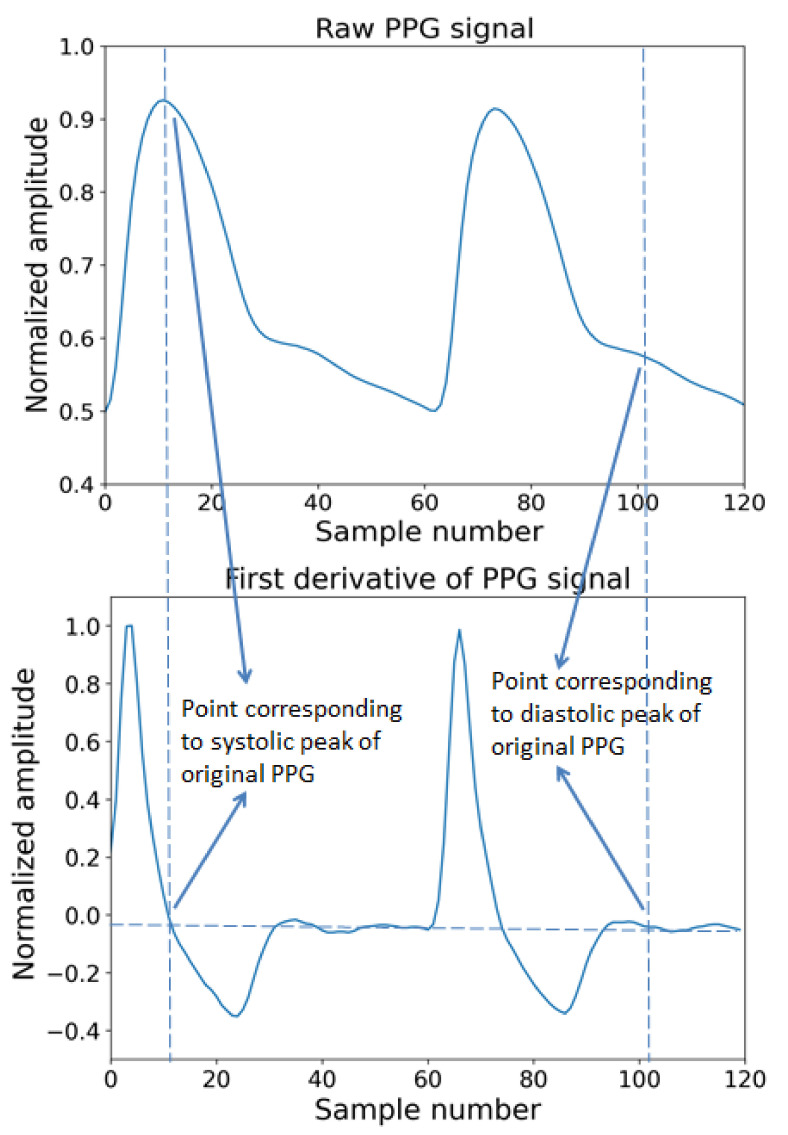
Original raw PPG wave and first derivative of PPG wave.

**Figure 6:**
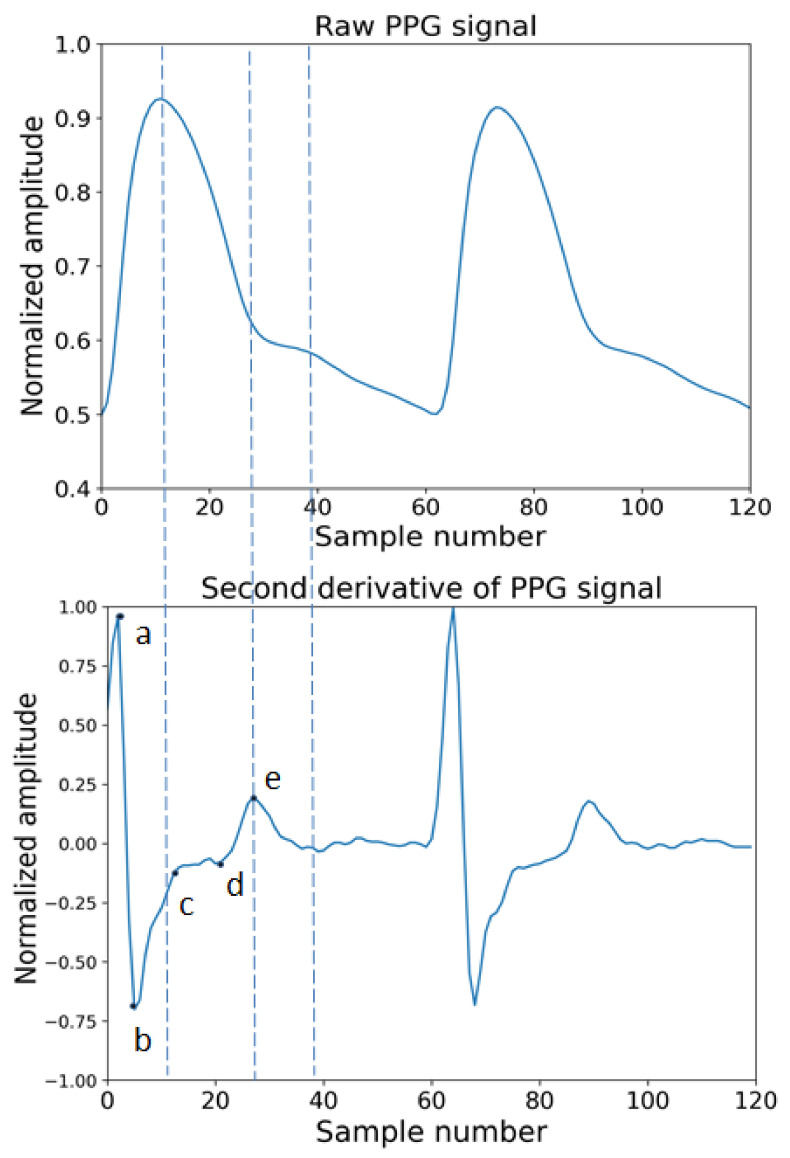
Original raw PPG wave and second derivative of PPG wave.

The derivatives of PPG waves are very useful for interpreting original PPG signals. In Figure 5, the original and the first derivative and Figure 6 shows a PPG signal and its second derivative. In Figure 5, the significant points in the first derivative were shown as ΔT and crest time (CT). The ΔT is the time interval between the first derivatives of PPG where the signal is going to zero from a positive direction to negative directions. The CT is defined as the time interval from the minimum of the PPG wave to its maximum peak. ΔT and CT are closely related to any abnormality related to a heart condition [85]. In Figure 6, the significant points are shown as points a, b, c, d, e. According to a previous study, the ratios, such as b/a, c/a, e/a, and (b-e)/a, etc., strongly related to the change in arterial stiffness [86]. The points a~e and the time interval between different points of ECG and PPG are used as features to measure blood pressure. 

### 2.3. Feature Extraction

**Figure 7:**
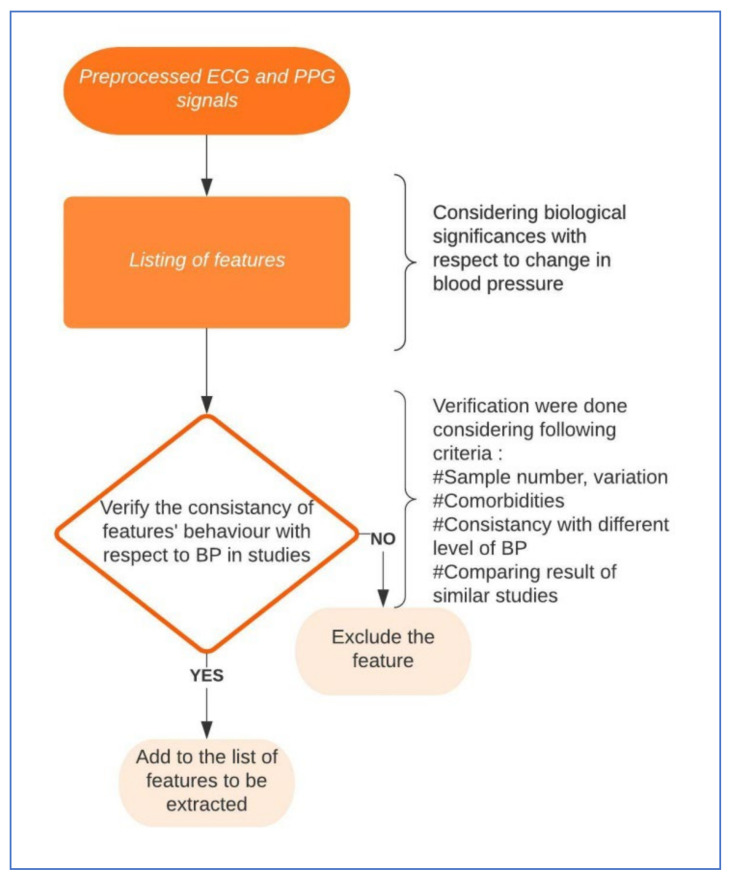
Diagram with the steps to finalize the features extraction process.

The whole process of finalizing the features to be extracted is shown in Figure 7. First, the morphological features from ECG will be discussed which are relevant to blood pressure measurement. The main challenge with ECG features is that the morphological changes in ECG for a hypertensive patient can either change for a short time period at the onset of high pressure or the change can sustain for a longer period [87]. The characteristics of the P wave from an ECG envelope, such as P wave area, amplitude, minimum duration, amount of dispersion, etc., have shown some relation with a change in blood pressure [88,89,90]. The length of PR interval increases with the increase of blood pressure [89,90,91,92]. The QT interval is the duration between Q waves until the end of the T wave, the characteristics which were found related to BP changes were: QT duration maximum, minimum, and QT dispersion [91]. The fragmented QRS complex is found more often with high blood pressure patients, also with a higher amplitude of the R and S wave. In the case of the T wave, the features are the same but the P wave was found to be related in some way to a change in blood pressure [90,93,94,95]. In previous studies, along with other discoveries, there were some shortcomings. For example, in some studies [91,92,94], the BMIs were not well spread out, some have patients mostly of a young age, some have limited patients with specific prior diagnosis, and several studies did not report any comorbidities or confounders, etc., [90,95]. These limitations require further study with these features before finalizing a specific list. Till today, there is no clear list of features from the ECG wave that are optimized in identifying hypertension reliably [96]. 

In the PPG wave, the first phase is related to the systolic period and the second phase is related to the diastolic period. Systolic amplitude is found to be suitable for measuring pulse arrival time [97]. The pulse width is the time duration for the whole pulse.

Researchers use different parts of pulse width for their experiment, such as using a half or quarter of the amplitude of systolic peak for their experiments [98]. Since the pulse width varies with the vascular resistance using the dicrotic notch, the area under the wave can be divided into two parts. The ratio of those parts indicates a strong relationship with total peripheral resistance, which is the force necessary to keep the blood flow from the aorta to the venous exit into the auricles [99]. An increase in total peripheral resistance will increase the blood pressure immediately. A recent study concluded that the correlation between PPG waveform and ABP waveform is very low. Therefore, the ABP cannot be replaced by the PPG wave for BP measurement.

They used synchronized signals, and a strong correlation indicated that their morphological changes are related and depend on each other; at the same time as the ABP increases, some different phases between the signals became visible. These changes have not been studied extensively yet so, although the morphological relationship between the signals shows a strong correlation with the increase of ABP, the change in correlation needs to be studied [100].

### 2.4. Feature Optimization and Selection

To identify the significant features from the available feature list, the feature selection and optimization algorithm need to be implemented. With the use of only relevant features the classification or data mining model ends up providing results with better accuracy and reducing computational complexity. A fundamental problem with any data is to approximate a relationship between input X = (x_1_, x_2_, x_3_… x_N_) and output Y = (y_1_, y_2_, y_3_,… y_N_). Sometimes, output Y cannot be determined by using all features but only a subset of features. If all the features including the irrelevant features were used there are a few problems that might occur. First, the irrelevant features will require more computational cost (sometimes polynomial greater) while doing any prediction. Secondly, the irrelevant features may produce overfitting which will fail the model when it is tested with any independent data from which it was trained on. In addition, when the mode is too simple and includes irrelevant features as well, under fitting occurs. That means it tends to have less variance in the prediction but more bias towards wrong outcomes [101]. The major advantages of optimal feature selection are: First, simpler models are simple to interpret. As the number of variables increases, the model gets more and more difficult to interpret. Second, the small number of features results in quicker training and prediction time. Third, redundant information from different features creates a false interpretation of higher accuracy but fails in the case of testing with data from a different dataset.

**Figure 8:**
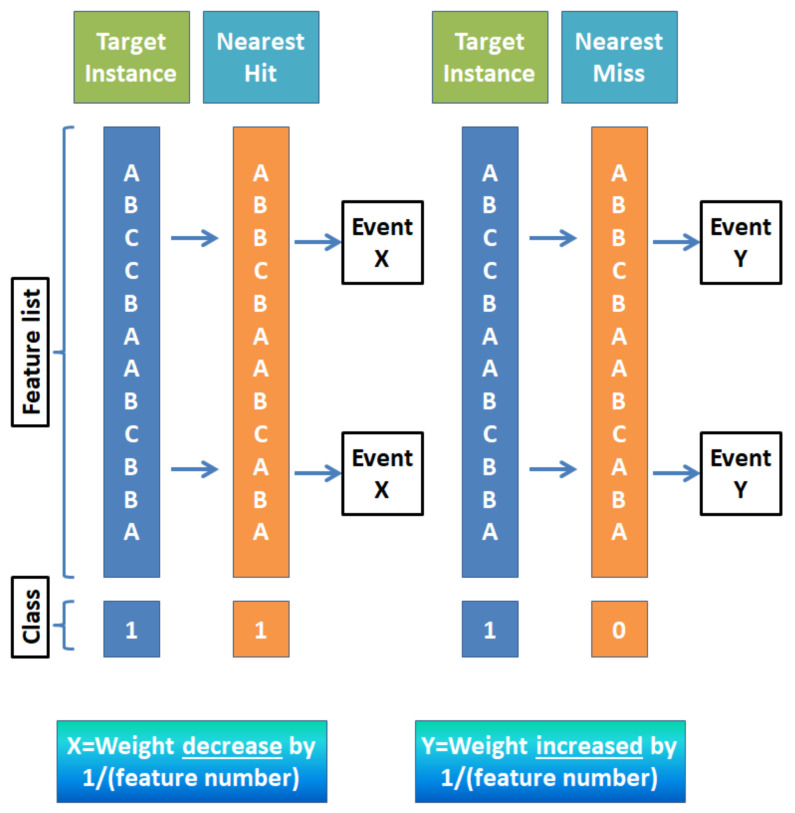
An example of feature weight update procedure based on distance target instance.

The filter-based method relies on feature characteristics and is favorable for the model which requires quicker selection. The wrapper method uses a machine-learning algorithm to select the best features in the subset and it is more computationally extensive than the filter-based method [102]. The embedded method is an iterative approach that extracts features that contribute most to the training for a particular iteration. In our study, the filter-based approach was primarily selected based on the following reasons. Unlike the wrapper-based method, the filter method does not incorporate any machine learning model to determine the relevancy of any feature. The filter method is much faster compared to the wrapper method because they do not require any training phase, so with a larger dataset, the difference in computation becomes quite large. Unlike the wrapper method, the filter-based method does not depend on a heuristic search algorithm. In the hybrid approach, the combination of filter and wrapper does not integrate well which may result in lower classification accuracy [103]. Popular filter-based feature selection methods use different techniques to differentiate between relevant and irrelevant features. For example: Euclidean/Manhattan distances between feature variables project dependence or correlation within features as well as between feature and target variable. An example of using Euclidean distance between features is the relief algorithm [104]. To better understand the aforementioned “distance” feature, Figure 8 is provided to introduce what is a nearest ‘hit’ or ‘miss’.

For a given target instance, which is shown in Figure 8, when it is compared with another instance with same class variable it is called a ‘hit’, and the other way around is called a ‘miss’. For a nearest hit and sample feature, such as A, B, C in Figure 8, when the values of the feature are different in those cases, the corresponding feature weight is decreased by 1/(total feature number). Similarly, in the case of a nearest miss for same scenario, weight for the corresponding feature is increased by same margin. An improved version of this technique involves calculating the distance function [105] of a specific feature (for example B) using the ratio of difference of value of that feature between target instance (TI) and the nearest hit/miss and the difference between the maximum of B and minimum of B.

The distance function is also used to find the nearest neighbors by calculating distance between different instances. The total distance is basically the summation of all the distance gathered from all features. An example of using dependence is Pearson or Chi-squared, where dependence or independence between two variables is measured [106]. Mutual information or information gain where information is shared between features or contributions of information of a feature are measured to differentiate between features [107].

In this paper, a combination of two feature selection methods is used. The first method is the fast correlation (FC) where it checks the correlation of each feature and relevance with the target variable. In 2021, it was discovered that FC has a problem of sometimes considering relevant features as redundant and ignoring them [108]. So, solely depending on FC cannot be the optimum choice. That is why the second method which is a ReliefF-based algorithm has been added in this work. It uses Manhattan distance instead of Euclidean distance in the Relief algorithm to differentiate between the same or different types of feature classes. ReliefF considers conditional dependencies or interactions between features which usually get ignored by the FC algorithm [109]. In both, the method of symmetric uncertainty was used as a measure of goodness for the features which is entropy-based correlation while correcting the bias of information gain. The reason behind choosing this over Pearson’s coefficient is the option to encompass the non-linear relationships between features since most physiological measurements are nonlinear [110]. In addition, the Pearson algorithm has very little robustness against outliers [111].

#### 2.4.1. Symmetric Uncertainty

To measure the feature effectiveness for classification, the feature has to be relevant to the class and at the same time not be redundant to any other relevant features. In this work, correlation is used as the criteria to select features. When the correlation between two features is small, but correlation with the target variable is higher than a certain threshold compared to another relevant feature, such a feature is selected as effective for that specific classification task. The linear correlation coefficient is as follows:

(1) $$r=\frac{\sum_{i}\left(x_{i}-avg(x_{i}\right))\left(y_{i}-avg\left(y_{i}\right)\right)}{\sqrt{\sum_{i}\left(x_{i}-avg(x_{i}\right){)}^{2}}\sqrt{\sum_{i}{\left(y_{i}-avg\left(y_{i}\right)\right)}^{2}}}\;$$

The mean r lies between −1 and 1 and represents the full correlation range between *X* and *Y*. When they are fully independent then the value of r will be zero. Correlation analysis removes the features which have a near-zero linear correlation with the class at the same time it reduces the redundancy between features considered linearly correlated. The limitation of linear correlation analysis is the assumption of linearity between features, which is not true for all features. In addition, all the features must only be in the numeric form to be considered for correlation analysis. The linear correlation coefficient assumes a linear relationship between variables even in cases where the relationship is quite non-linear, so just by getting a low or near-zero Pearson’s correlation coefficient it is difficult to correctly conclude that they are independent of each other.

To solve these shortcomings, a potential solution is to choose a correlation measure based on entropy and use mutual information (MI), which is the reduction in uncertainty of one variable while observing the other variable. If we compare how the joint distribution of the variables differs from the multiplication of their marginal distribution, it can be concluded that they have dependence whether they are linear or nonlinear [112]. A discrete random variable X with values {*x*_1_, … … *x_k_*}. The entropy for the variable X is H which measures the uncertainty of the prediction of X [113]. So, the entropy is defined as:

(2) $$H\left(X\right)=-\sum_{i}P\left(x_{i}\right)log_{2}(P\left(x_{i}\right))\;$$

For *x_i_*, the prior probability is *p*(*x_i_*), and the entropy *H*(*X*). *H*(*X*|*Y*) can be expressed as follows [114]:

(3) $$H\left(X|Y\right)=-\sum_{j}[\;P\left(x_{i}\right)]\sum_{i}P\left(x_{i}|y_{j}\right)log_{2}(P\left(x_{i}|y_{j}\right))\;$$

Now the mutual information can be introduced which measures the reduction in the uncertainty of *X*.

(4) $$\;MI\left(X|Y\right)=H\left(X\right)-H\;$$

In case of X and Y, they are independent of each other, *H*(*X*,*Y*) = *H*(*X*) + *H*(*Y*), *M*(*X*|*Y*) = 0 and *H*(*Y*|*X*) = *H*(*Y*). In contrast, when *X* and *Y* are fully correlated, the joint entropy *H*(*X*|*Y*) = 0 and mutual information *M*(*X*|*Y*) = *H*(*X*). Although symmetry is a desirable property when we want to consider it to be an effective measure for features, MI or information gain (IG) are biased towards high cardinality features. So, *MI* or *IG* needs to be normalized using the entropies of the features *H*(*X*) and *H*(*Y*) to compensate for such bias. The resultant correlation measure is called symmetrical uncertainty (*SU*) [115], and it is given by

(5) $$SU\left(X,Y\right)=2*\frac{Mutual\;information}{H\left(X\right)+H\left(Y\right)}\;\;$$

The range of *SU* is between 0 and 1, where the value 0 indicates that *X* and *Y* are independent. Even with *SU*, there exists a limitation of computing more than two variables. So, an extension is necessary for the multivariable cases to detect interactions among different sets of variables. 

Before moving towards multivariable symmetric uncertainty (MSU), let us first discuss the concept of total correlation [116]. For a given vector of variables X, the joint entropy for *n* number of variables is H(*X*_1:*n*_), given by [114]

(6) $$H\left(X_{1:n}\right)=-\sum_{x_{1}}\ldots \sum_{x_{n}}P\left(x_{1},\ldots ,x_{n}\right)log_{2}[P\left(x_{1}\ldots ,x_{n}\right)]\;$$

For *n* number of variables, the mutual information can be expressed as a reduction of the uncertainty among one or more variables by knowing another. It is a comprehensive measure of independence, which means when the variables are independent then joint entropy equals the sum of their marginal entropies. When it is not equal then some sort of dependency is present. That is why it is better to consider the linear and nonlinear dependencies compared to linear correlation [117]. For *n* number of variables, the total correlation is shown in Equation (7). Here, total correlation does not indicate only linear correlation but linear and nonlinear dependencies, thus giving total mutual information:

(7) $$C\left(X_{1:n}\right)=\sum_{i=1}^{n}H\left(X_{i}\right)-H\left(X_{i:n}\right)\;$$

If we use *n* = 2, then the total correlation will turn into information gain or mutual information between the two variables.

(8) $$\begin{array}{ll} C\left(X_{1:2}\right) & =C\left(X_{1},X_{2}\right)\\ & =\sum_{i=1}^{2}H\left(X_{1}\right)-H\left(X_{1:2}\right)=H\left(X_{1}\right)+H\left(X_{2}\right)-H\left(X_{1},X_{2}\right)\\ & =H\left(X_{2}\right)-H\left(X_{2}|X_{1}\right)=H\left(X_{1}\right)-H\left(X_{1}|X_{2}\right) \end{array}$$

(9) $$=H\left(X_{1},X_{2}\right)-H\left(X_{2}|X_{1}\right)-H\left(X_{1}|X_{2}\right)\;=-\sum_{X_{1},X_{2}}P(X_{1},X_{2})log\left(X_{1},X_{2}\right)+\sum_{X_{1},X_{2}}P(X_{1},X_{2})log\frac{P\left(X_{1},X_{2}\right)}{P\left(X_{1}\right)}+\;\sum_{X_{1},X_{2}}P(X_{1},X_{2})log\frac{P\left(X_{1},X_{2}\right)}{P\left(X_{2}\right)}\;\;=\sum_{X_{1},X_{2}}P(X_{1},X_{2})log\frac{P\left(X_{1},X_{2}\right)}{P\left(X_{1}\right)P\left(X_{2}\right)}\;=I\left(X_{1},X_{2}\right)\;$$

Zero or close to zero value of *C*(*X*) means that all the variables are independent of each other. To transfer the concept of total correlations into multivariable symmetric uncertainty (MSU) keep the total correlation or total mutual information *C* between 0 and 1 and recognize that a higher value of *MSU* should indicate a higher correlation among variables. Equation (4) is the mutual information between two variables and for several *n* variables according to Equation (7), the total mutual information can be expressed as: $\sum_{i=1}^{n}H\left(X_{i}\right)-H\left(X_{i:n}\right)$. By definition, the symmetric uncertainty is the normalized mutual information, so the denominator of Equation (5) becomes the summation of entropy of all variables which is equal to: $\sum_{i=1}^{n}H\left(X_{i}\right)$. The symmetric uncertainty of two variables from Equation (5) becomes the following equation as the general formula for *MSU* where value 2 in the case of two variables is expressed as a normalization multiplier in the case of *MSU*:

(10) $$MSU\left(X_{1:n}\right)=normalization\;multiplier\left(m\right)*\frac{\sum_{i=1}^{n}H\left(X_{i}\right)-H\left(X_{i:n}\right)}{\sum_{i=1}^{n}H\left(X_{i}\right)}\;=m*[1-\frac{H\left(X_{i:n}\right)}{\sum_{i=1}^{n}H\left(X_{i}\right)}\;$$

To prove Equation (10), using the chain rule [118],

$$H\left(X_{i:n}\right)=\sum_{i=1}^{n}H\left(X_{i}|X_{1,\ldots ,\;}X_{i-1}\right)=\sum_{i=1}^{1}H\left(X_{i}|X_{1,\ldots ,\;}X_{i-1}\right)+\sum_{i=2}^{n}H\left(X_{i}|X_{1,\ldots ,\;}X_{i-1}\right)\;\;=H\left(X_{1}\right)+\sum_{i=2}^{n}H\left(X_{i}|X_{1,\ldots ,\;}X_{i-1}\right)$$

This proves that $H\left(X_{i:n}\right)\geq \;H\left(X_{1}\right)\;\mathrm{or}\;\mathrm{in}\;\mathrm{general}\;H\left(X_{i:n}\right)\geq \;H\left(X_{i}\right)$.

So, (a) $n*H\left(X_{i:n}\right)\geq \;\sum_{i=1}^{n}H\left(X_{i}\right)$.

In addition, similarly, $H\left(X_{i}|X_{1,\ldots \ldots ,\;}X_{i-1}\right)\;\leq H\left(X_{i}\right)$.

This result: $H\left(X_{1}\right)+H\left(X_{2}\right)+\cdots +H\left(X_{n}\right)\geq \;H\left(X_{i:n}\right)$.

Or (b) $\sum_{i=1}^{n}H\left(X_{i}\right)\geq \;H\left(X_{i:n}\right)$.

Using the inequality of (a) and (b),

$$\frac{1}{n}\leq \frac{H\left(X_{i:n}\right)}{\sum_{i=1}^{n}H\left(X_{i}\right)}\leq 1$$

Since the MSU in Equation (10) goes from 0 to 1, using the above right-side limit of $\frac{H\left(X_{i:n}\right)}{\sum_{i=1}^{n}H\left(X_{i}\right)}$ and definition of MSU,

$$MSU\left(X_{1:n}\right)=m*\left[1-\frac{H\left(X_{i:n}\right)}{\sum_{i=1}^{n}H\left(X_{i}\right)}\right]=m*\frac{n-1}{n}=1,$$

So, $m=\frac{n-1}{n}$ MSU can be reduced to:

(11) $$MSU\left(X_{1:n}\right)=\left(\frac{n}{n-1}\right)*\left[1-\frac{H\left(X_{i:n}\right)}{\sum_{i=1}^{n}H\left(X_{i}\right)}\right]$$

Here the nm represents the normalization multiplier; from [119] the nm was be defined as *n*/(*n* − 1). In the case of two variables, the value of nm was 2. To prove that SU and MSU are the same measures, it can be derived that when *n* = 2 the MSU will become SU.

(12) $$MSU\left(X_{1:2}\right)=\left(\frac{2}{2-1}\right)*\left[1-\frac{H\left(X_{i:2}\right)}{\sum_{i=1}^{2}H\left(X_{i}\right)}\right]\;\;\;\;\;\;=2\frac{H\left(X_{1}\right)+H\left(X_{2}\right)-\left[H\left(X_{1}\right)+H\left(X_{2}|X_{1}\right)\right]}{H\left(X_{1}\right)+H\left(X_{2}\right)}\;=2\frac{H\left(X_{2}\right)-H\left(X_{2}|X_{1}\right)}{H\left(X_{1}\right)+H\left(X_{2}\right)}=SU\left(X_{2},X_{1}\right)=SU\left(X_{1},X_{2}\right)$$

#### 2.4.2. Fast Correlation Algorithm Using MSU

In this research, the fast correlation method which was introduced in 2004 is combined with MSU to perform the calculation of feature selection. The algorithm works in two steps. In the first step, the features are sorted using MSU, and a threshold setting is used to remove less correlated features. In the second step, the comparisons among the features were done to remove the redundant features using MSU. So, this algorithm uses the accelerated pace advantage of filter and achieves higher efficiency of calculation at the same time.

**Figure 9:**
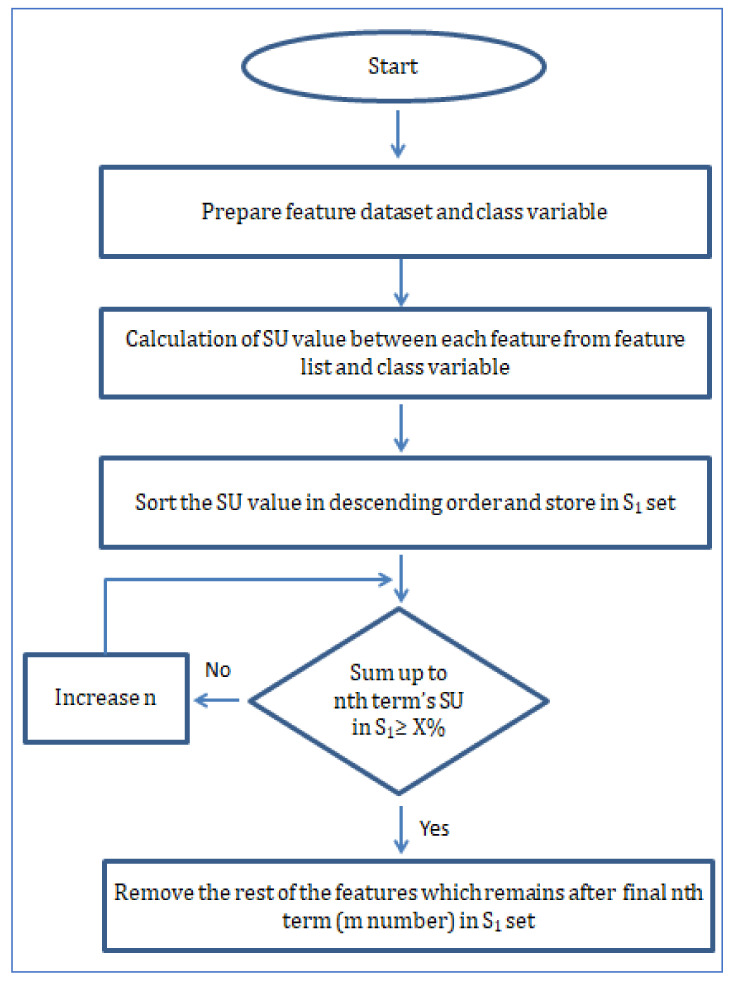
Step 1: Flowchart of fast correlation using SU value to find the relevant feature set.

**Figure 10:**
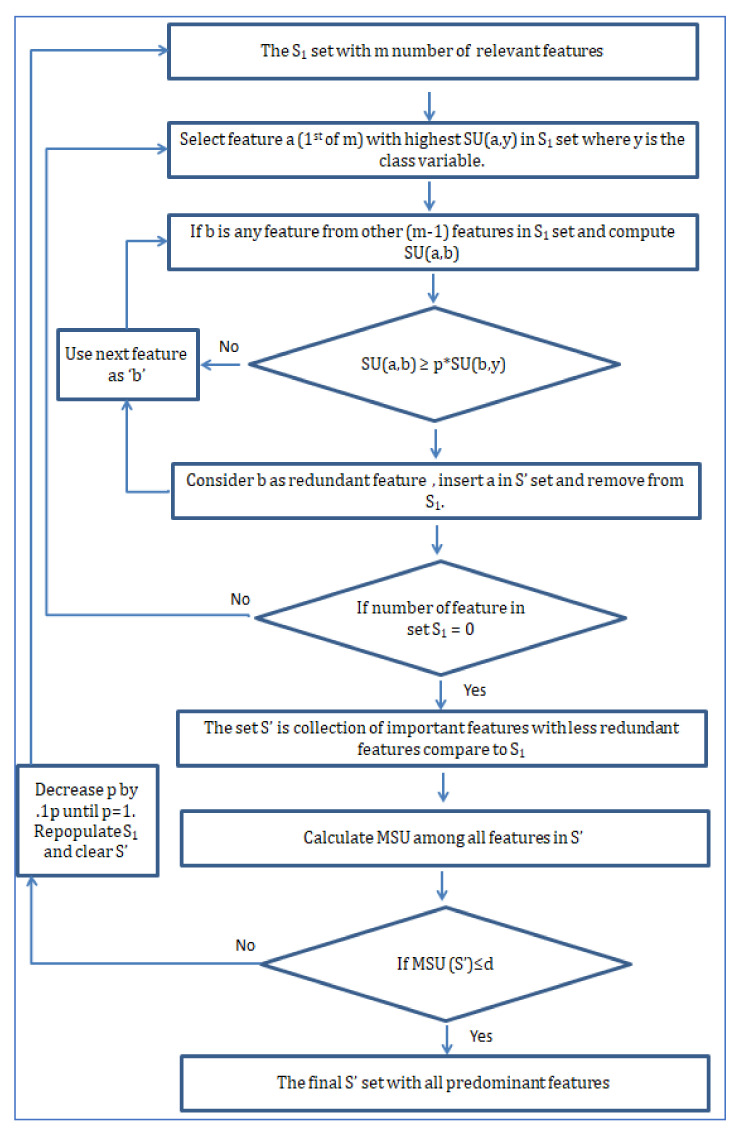
Step 2: Flowchart of fast correlation using SU and MSU to remove redundant features.

Here the two steps of the algorithm are shown in two separate flowcharts. The first flowchart in Figure 9 shows how the relevance of features is measured, and in the second flowchart in Figure 10, the process of removing redundant features is shown.

Here in Figure 9, the diagram of step 1 of the algorithm has been depicted. The majority of step 1 involves calculating SU values between features and class variables to make a ranking list. Based on the predefined high threshold, the selected features are put into a set called S_1_ as a collection of strong relevant features with class variables. Although the S_1_ set includes features that show a strong relation with the class variable at the same time, it does not confirm whether there are any redundant features present or not in set S_1_. For that purpose, step 2 of the algorithm is depicted in Figure 10. The main objective of step 2 is to remove redundant features from S_1_ and only keep the predominant features. When a feature that is ranked high in set S_1_ has a higher SU with the next feature in set S_1_ than the SU between the next feature and class variable, we can safely confirm the next feature as redundant. Using this principle, all the features in S_1_ are used in the same calculation pattern and marked as either redundant or not. While making the comparison using SU, a constant p is included to have a final list with a varied number of features.

Based on the MSU of the feature list in S’ after each round, the value of p will be reassigned (decreased by 0.1 *p* until *p* = 1) to make the feature number in S’ smaller. Finally, after achieving a specific near-zero value of MSU, this points to an S’ which consists of only predominant features. 

#### 2.4.3. Relief Algorithm 

Most of the feature selection algorithms assume the conditional independence of features (dependent on target variable but not on each other), these algorithms are not appropriate for solving a problem where there are many feature interactions. Relief algorithms do not assume so the quality of attributes can still be extracted with high interaction among them. The main idea is how their contribution and separation patterns behave when they are close to each other in different instances [105]. Depending on whether the random instance *X*_I_ and H result in different values for the F feature then *F* identifies them as belonging to the same class, but this scenario is not desirable, so the weight of *F* is reduced. On the other hand, *X*_I_ and M result in different values for the *F* feature, then F identifies them as the same class, but this scenario is desirable, so the weight of F is incremented. Let us assume the whole instance list is *Y*_1_, *Y*_2_, *Y*_3_,…*Y_a_* which are used to describe a set F of attributes *F* = 1, 2, 3,…*n*, where *n* is the number of features. So, the function can be expressed as below for nominal features [120]:

(13) $$diff\left(F,Y_{1},Y_{2}\right)=\left\{\begin{array}{c} \;0\;,\;when\;value\;\left(F,Y_{1}\right)=\left(F,Y_{1}\right)\;\\ 1,\;otherwise \end{array}\right.$$

For numeric features the difference function becomes as below [120]:

(14) $$diff\left(F,Y_{1},Y_{2}\right)=\frac{\left|value\left(F,Y_{1}\right)-value\left(F,Y_{2}\right)\right|}{Max\left(F\right)-min\left(F\right)}$$

Regarding limitations, research in a Relief-based algorithm has been limited to two-way interactions only [120]. It has been proved in several types of research that empirically the core performance of the Relief algorithm deteriorates as the number of irrelevant features increases, so before using the feature through a Relief-based algorithm it is beneficial to pass them through another process to decrease the number of irrelevant ones [121]. In addition, the Relief algorithm cannot work with multiclass endpoints. So, there must be an improved algorithm that can provide the advantage of Relief and solve the limitations mentioned before.

#### 2.4.4. ReliefF with MSU

ReliefF is the best-known Relief-based algorithm [104] which is the fixed algorithm variation (A to F). There are four key aspects to the improvement in the ReliefF algorithm. First, ReliefF uses the user-defined K number of neighbors and updates in the Relief algorithm [122]. Second, in ReliefD (which is also incorporated in ReliefF) the best solution to encounter missing data was proposed by equating diff function to probability that for a given feature two instances do not show the same value [123]. Third, ReliefF proposes an approach to handling multi-class target points where ReliefF calculates the prior probability from class while finding k nearest misses. Fourth, the reliability of weight estimation becomes credible as m becomes close to *n* [105].

The most important step in the ReliefF algorithm is after calculating the diff function, the estimation of weight for each feature, which is [124]

(15) $$W\left[F\right]≔W\left[F\right]-\frac{\sum_{j=1}^{k}diff\left(F,Y_{i},Y_{j}\right)}{n.k}+\sum_{C\;\neq \;class\;\left(Y_{i}\right)}\frac{p\left(C\right)}{1-p\left(class\left(Y_{i}\right)\right)}*\frac{\sum_{j=1}^{k}diff\left(F,Y_{i},Y_{j}\left(C\right)\right)}{n.k}$$

*Y_i_* is a randomly picked sample at time *t_i_*; the nearest number of neighbors of *Y_i_* is *k*. Although the Relief algorithm has advantages such as being non-myopic, its ability to estimate the quality of features requires less time than the exhaustive search approach. Its main limitation is the inability to consider feature interactions for more than two features [125].

The ReliefF algorithm proposes a correlation between the calculation pattern and each fault category. The objective is to find K nearest neighbor samples from the sample set. The other way around is called near hits. In this paper, the MSU is used to create a ranking of features based on symmetric uncertainty and use the ranking for the next steps. This will reduce the computational complexity at the feature weight measurement step compared to the use of every feature in that step.

### 2.5. Regularized Regression with a Penalty Factor

In this section, the necessity of regularization in regression technique will be discussed along with the lasso, ridge, and combined effect of those two regression techniques. Since the result of the techniques discussed in the previous subsection was to optimize the number of features, this subsection will discuss the regularized regression technique which will be implemented using those features to get a cuffless measurement method of blood pressure. The basic linear regression model predicts *n* observations of target variable Y using the linear combination of m predictor variable X. If the error term is normally distributed with variation *σ*^2^:

(16) Y=Xβ+ϵ

In the ordinary least square (OLS) approach, the β is estimated in such a way that the sum of squares of residuals is as small as possible. So, the objective is the minimizing loss function:

(17) $$minimize\;\{SSE=\sum_{i=1}^{n}{\left[y_{i}-y_{i}\left(mean\right)\right]}^{2}={\left|\left|y-X\beta_{est}\right|\right|}^{2}\}$$

In statistics, two major components to measure the characteristics of estimators or predictors are bias and variance. The bias is the difference between the estimated target variable and true output variable and bias mainly measures the accuracy of the prediction. On the other hand, variance calculates the variation or spread or uncertainty of the predictions and the unknown error variance which can be expressed as below [126]:

(18) $$error\;variance=\frac{\left(y-X\beta_{est}\right)\left(y-X\beta \right)}{n-m}$$

The expectation of OLS is low bias and variance since large values indicate poor performance of the OLS approach. The total error from the model can be categorized into three segments as follow [126]:

(19) $$Model\;error=error\;from\;bias+error\;from\;variance\;+unexplained\;error{\left(\sigma^{2}\right)}_{\;}$$

Based on whether the value for bias or variance is high or low there can be four different situations. The high value for variance and bias provides the worst prediction and, on the other hand, low value of variance and bias provides the best performance. The OLS generally provides a solution where it is unbiased, but the variance is high, so for this specific situation three problems need to be solved. They are multicollinearity, interpretability, and insufficient solution. Finally, the third issue is the balance between variance and bias (interpretability). If the model complexity and error terms are plotted, as the model complexity (number of features) increases three things happen: first, error due to bias decreases sharply; second, error due to variance increases. The total error starts high with a low number of features; it comes down to the lowest value for a certain number of features and then grows large with more features in the model. So, keeping increasing the feature number is not the solution, but the objective should be to find the optimized situation with the right balance between bias and variance. A solution for all these constraints is adding a regularization term or penalty term with OLS which will lower the variance at the cost of some bias to find the optimum solution. 

When there are many predicting variables with small effects from each of them which are correlated among each other, ridge-type regression can prevent the linear regression model from showing poor performance (high variance) [127]. In ridge regression, not only does the OLS loss function get minimized, but also the size of parameter estimates gets penalized too as shown in Equation (20) [127].

(20) $$minimize\;\{SSE+lambda\sum_{j=1}^{p}\beta_{j}^{2}\}$$

The lambda (*λ*) is the regularization penalty. The lasso regression technique is used for large datasets. The change from ridge regression to lasso is the penalty term as follows:

(21) $$minimize\;\{SSE+lambda\sum_{j=1}^{p}\left|\beta_{j}\right|\}$$

The major shortcoming of the lasso technique is oracle property and instability with high-dimension data [128]. Although both techniques use correlated predictors, they solve the multicollinearity differently. In ridge regression, the coefficients of correlated predictors are reduced but remain like each other, and in lasso regression, the correlated predictors end up with a large value of coefficient while the rest of the predictors are zeroed. Since it is not possible to determine true optimized parameter values, there has to be a way to use the penalty term in a way to optimize the coefficient values. 

To take advantage of both the ridge and lasso methods, a hybrid approach can be to use a combination of them as follows:

(22) $$minimize\;\{SSE+lambda_{2}*\alpha *\sum_{j=1}^{p}\left|\beta_{j}\right|+lambda_{1}*\frac{1-\alpha }{2}*\sum_{j=1}^{p}\beta_{j}^{2}\}$$

Here, the value for α makes the choice between ridge and lasso technique, where α=0 means ridge regression and α=1 means lasso regression. There is an extra quadratic term that results in a convex loss function. The inclusion of a little bias while reducing the variation is the appropriate solution. In lasso, it is a possibility that some of the features will be pushed towards zero and even totally zero, so ridge regression is more effective since it does not drastically reduce the feature number but rather takes a systemic approach of reducing feature weight. The following Figure shows the change in number and value of coefficients lambda and alpha in different situations for a sample scenario.

When alpha is equal to one, the elastic net becomes lasso regression, and the number of coefficients becomes smaller while the value of lambda gets larger. On the other hand, when the value of alpha is equal to zero, the elastic net becomes ridge regression, and the number of coefficients remains the same for different values of lambda indicating that ridge does not reduce the number of a predictor. So, when the value of alpha is between zero and one the pattern in which the value of coefficient and number of coefficient changes differs from ridge and lasso. To get the optimum number of features the implementation of cross-validation needs to be used. From the mean square error versus lambda plot, the number of the coefficient which will keep the MSE smallest for a certain value of lambda can be achieved.

## 3. Data Analysis

In this section, the dataset which has been used in this paper is introduced and later the data analysis process will be described.

### 3.1. Dataset

A dataset called “MIMIC II” used in this research is available on the physionet website [129]. From intensive care patients at the hospital, several vital signs were measured synchronously for a specific time frame, the vital signs which were recorded are ECG, arterial blood pressure, and fingertip pulse photoplethysmography. Between 2001 and 2008, a total of 26,870 patients were admitted in ICU. The dataset can be divided into two sections, the first one is clinical data and the second one is the physiological waveform of the measured vital signs. The waveform was collected as a raw dump file where there is some separate file for each patient and at the same time some files contain more than one patient due to the machine not being reset between patients. Those cases were separated by manually checking for the exceptional long intervals between readings. Although the initial recording of ECG was done in 12-bit precision and a higher sampling rate, later for the ease of calculation it was reduced to 7–10 bits. Later “peak picking” techniques were used to scale down the sampling rate to 125 by choosing every one peak from four consecutive ones.

**Figure 11:**
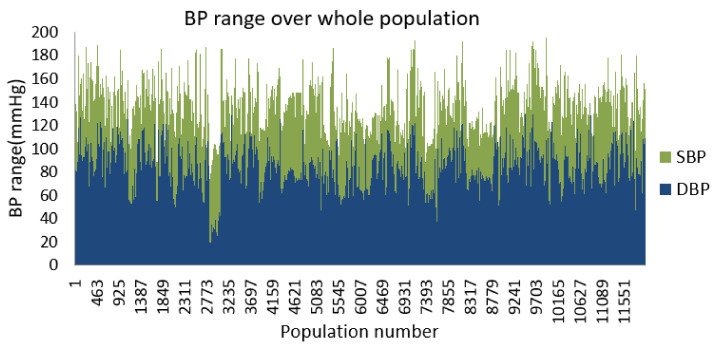
The range of blood pressure over all populations.

Although the data originally was from the physionet website, it was sampled at 125 Hz and stored as MATLAB files in the UCI repository [23]. The total dump file is divided into three separate MATLAB data files, each containing 3000 admission scenarios with ECG, PPG, and ABP data in separate rows. So, in this research, the first 1000 samples were considered to make it homogeneous. In Figure 11, the distribution of blood pressure over a total population of 12,000 is shown.

**Figure 12:**
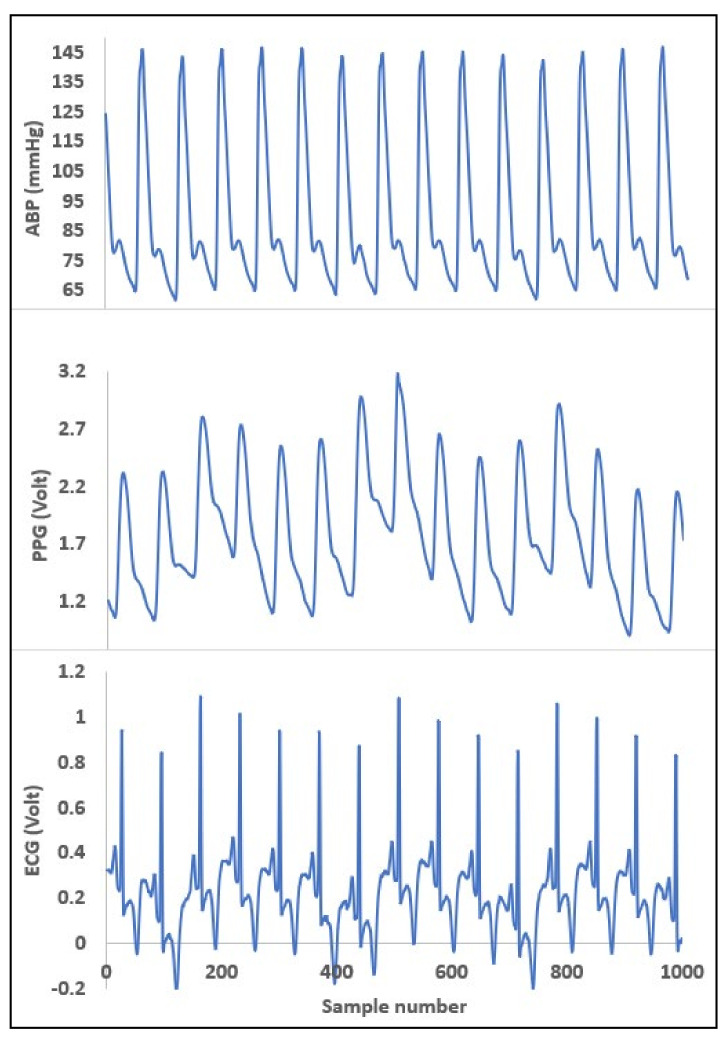
Example of a raw data from a patient with first 1000 samples.

Also in Figure 12, an example of raw data representation from a patient of three vital signs is shown.

### 3.2. Data Analysis

The raw signals from the dataset contain the three attributes but along with the signals, there were also some unwanted artifacts present. 

Before removing unwanted noises, the dataset was manually checked for missing, outlier, or unusually different data points due to possible anomalies in the measurement system. From the 12,000-admission dataset of three attributes, the selected number of data was 3295. In the previously mentioned steps in the pre-processing subsection, the section was used to get rid of the noises and prepare the signal data ready for the data extraction phase. Since the FIR filter is linear but ECG is a non-linear signal, there is a possibility that there would be some delay involved as a result [130]. There were delays observed due to the use of the FIR filter. Using Fs and filter order, the delay comes as 0.196 s. If we had decided to increase the order of the filter, the time delay would have increased, which is a disadvantage of the FIR filter. Although this time delay did not hamper the calculation of heart rate or R peak detection but considering any other heart condition other than heart rate variability, we had to fix the delay. So, using the FIR filter, the delay does not distort the signal waveform, and it is possible to compensate for the delay simply by shifting the filtered signal by a specific sample to match the filtered version of the original signal waveform. 

**Figure 13:**
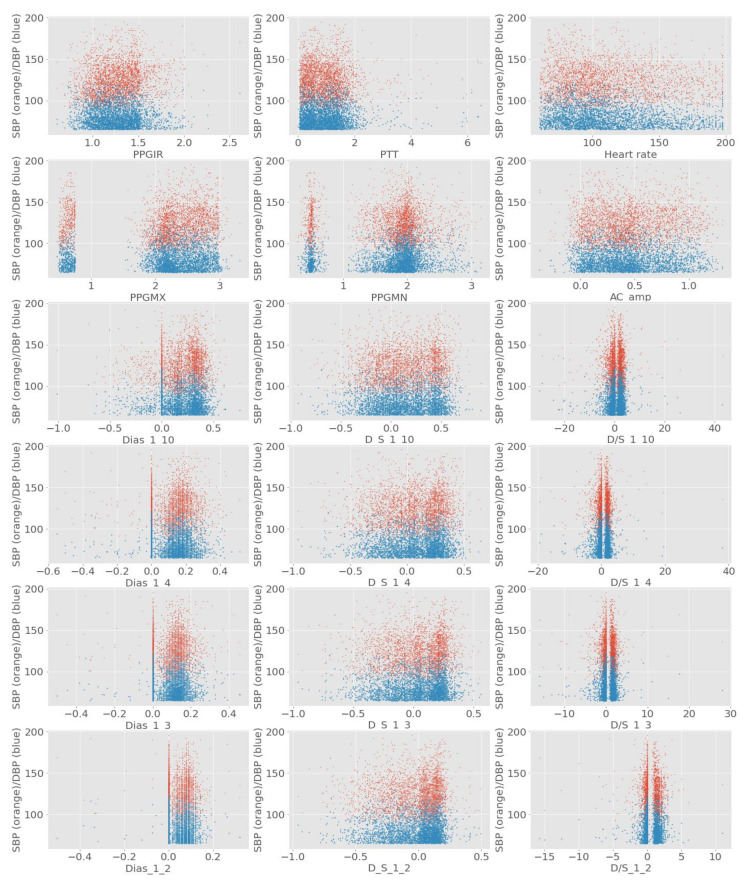
The distribution of SBP and DBP against some of the extracted features.

In the feature extraction phase, importance was given to the feature’s biological significance regarding a change in blood pressure. The initial list of features to be extracted mostly consisted of different time intervals and amplitudes of specific wave sections of ECG and PPG waves. In Table 1, the detailed lists of features were given. The goal of the feature selection method was to use linearly and nonlinearly correlated features to be prioritized. Initially, the ranking using Pearson’s linear correlation coefficient is shown in Table 2. In addition, in Figure 13 some of the distributions of the extracted features against their respective SBP and DBP values are depicted. 

**Figure 14:**
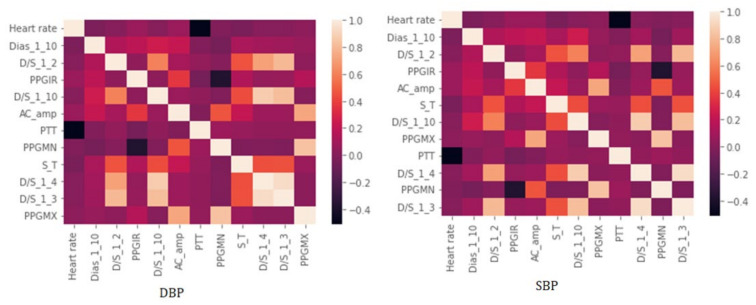
The Pearson’s coefficient value among finalized features using a heat map.

The implementation of fast correlation using MSU and ReliefF-MSU algorithms was done using python language. The top 12 features were finalized based on the combined ranking of feature selection algorithms which were implemented based on the main rule of maximizing the relevance with the target variable but reducing the number of redundant features at the same time. It was found that using the proposed technique reducing the feature number to less than 12 produced results where the accuracy was less than the intended margin. In Figure 14, the heat map for the finalized feature for SBP and DBP is shown. Although the heat map only considers the linearly correlated coefficient and does not represent the correlation in terms of nonlinearly correlated features, it is evident from the heat map that even from the linear perspective the correlations among finalized features are quite small.

**Figure 15:**
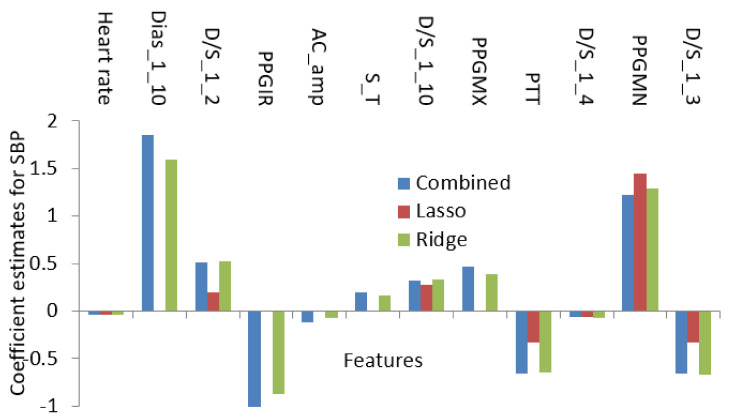
The value of coefficient for penalty-based regression for SBP.

**Figure 16:**
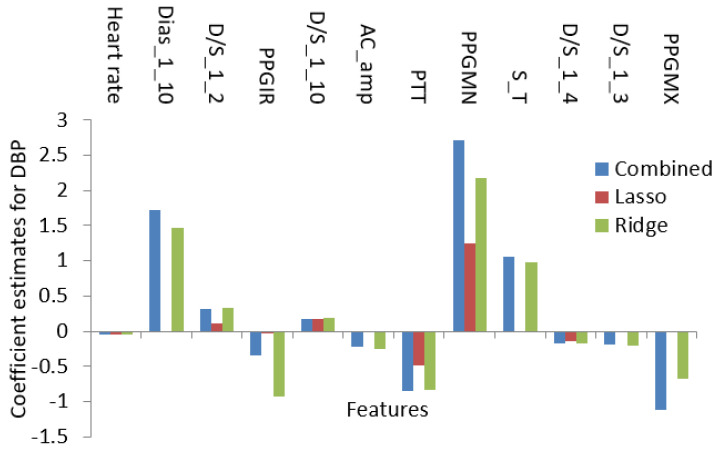
The value of the coefficient for penalty-based regression for DBP.

In this paper, the regularized techniques were applied because they keep the variance in control and bring a balance between bias–variance convex relationships. In addition, by nature regularization favors simpler models by addressing issues like variance-bias trade-off, multicollinearity, sparse data handling, and partly feature selection. Comparing popular models such as lasso and ridge where lasso assigns a penalty to reduce the number of variables and ridge assigns a penalty to reduce the impact of overemphasized variables, lasso showed some inconsistency due to the presence of multicollinearity even in smaller degree and ridge showed some inconsistency due to the lack of ability to reduce any variable to zero even when the situation required it. So, there must be a trade-off between the penalties coming from both lasso and ridge regression to make it balanced. The result of the processes which are coefficients or weights of estimates is shown in Figure 15 and Figure 16.

Using the features in Table 1 as the initial set, the optimization algorithms produced a result with the features listed in Table 2. The features in Table 2 encompass both linear and nonlinear relationships and using only those features the regression analysis was conducted. So, Figure 15 and Figure 16 contain the features which not only have shown linear dependence but also from the aspects of both linear and nonlinear relationships.

**Figure 17:**
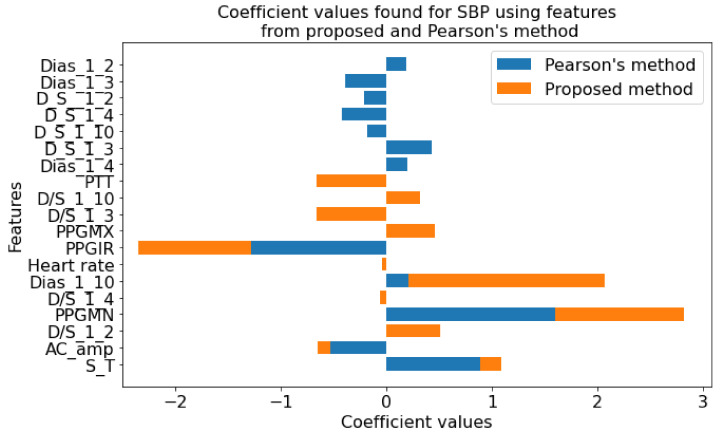
Coefficient values found for SBP using features from proposed method and Pearson’s method.

**Figure 18:**
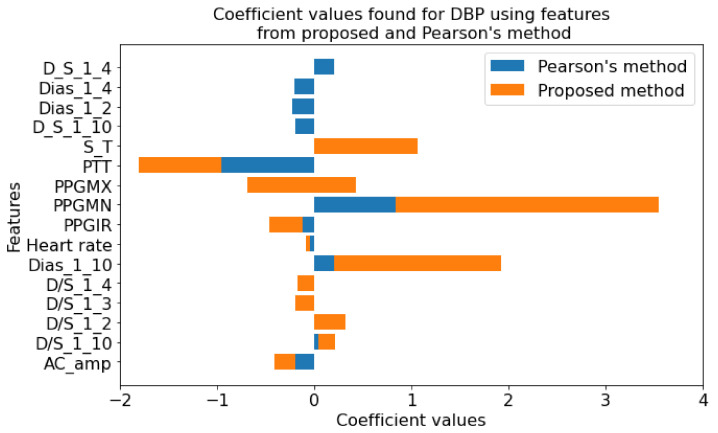
Coefficient values found for DBP using features from proposed method and Pearson’s method.

In Figure 17, the coefficient values for features from the proposed method and Pearson’s method are shown. Few features were found common in both methods. However, more than half of the numbers of features are different. This variation in the selection of features proves the fact that using the linear relationship will only result in missing some more relevant features. For example, although in many studies the linear relationship between pulse transit time (PTT) and blood pressure was assumed, recently it has been proven that they are nonlinearly related [131]. In this study for SBP, the coefficient value is zero for the PPT feature when we use Pearson’s method. However, in the proposed method, the PTT coefficient is relatively significant confirming the findings. Similar conclusions can be drawn regarding DBP, as shown in Figure 18.

## 4. Result and Performance Evaluation

**Figure 19:**
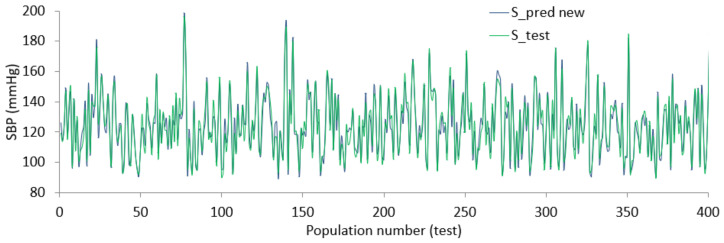
Predicted value against actual value for SBP.

**Figure 20:**
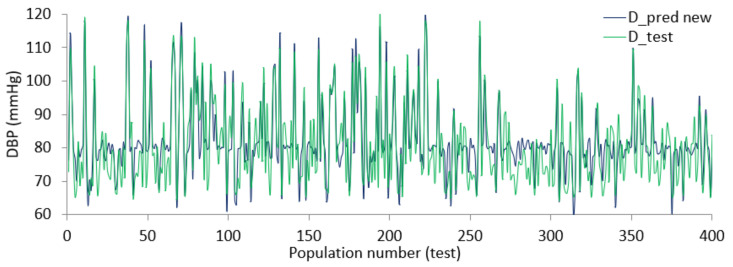
Predicted value against actual value for DBP.

Using the coefficient values from the combination of ridge and lasso regression, the final relation between finalized features and SBP or DBP is defined. The regularized BP models were tested using data from the dataset resulting in the diagrams shown in Figure 19 and Figure 20. From the Figures, there is evidence that the predicted SBD and DBP closely follow the test value in most cases.

The error varies within (+/−) 15 mmHg in a worst-case scenario, for more than half of the test points the error remains within (+/−) 5 mmHg. The size of the dataset was large and although some parts of the dataset were ignored due to lack of records or inconsistent values, the performance shown by regularized regression using an optimized feature list was still promising. Since each of the feature selection algorithms works from different aspects, the outcome of the algorithms provides an optimized feature list. In addition, since the objective of this study is to bring forth a generalized relation and not a person-specific one, it was expected that there would be some discrepancies between the predicted value and test value.

The summary of the study in terms of root mean square error (RMSE) and mean absolute error (MAE) and the comparison with similar studies, where feature optimization and regression relations were used, are depicted in Table 3. Using the proposed technique, the performance indices MAE and RMSE improved significantly compared to the Pearson-based method. This performance improvement is the result of the inclusion of both linear and nonlinear relationships in the feature selection algorithm and the use of a penalty-based regression algorithm. The MAE and RSME for SBP were better than those of DBP for the proposed method. The main reason behind this difference is that nonlinearity of peripheral arterial compliance affects the dependency of PTT and SBP more than DBP [132]. In addition, by measuring hemodynamic parameters such as stroke volume and total peripheral resistance another study proved that the linear correlation is higher for DBP compared to SBP with pulse wave velocity and arterial stiffness parameters [133]. So, since the proposed method took linear and nonlinear relationships into account, the amount of improvement happened more for SBP than DBP. The number of features replaced from Pearson’s method to the proposed method also confirmed that for SBP there was a higher number of features needed to be replaced than DBP.

The value of MAE from the proposed method resulted slightly higher than the values from studies [134,135]. The experimental model of [136] was prepared using factors such as viscosity of arterial wall and pulse wave reflection but their number of subjects and age range were extremely small. They have used only 16 subjects with an age range of 19 to 25 only which lessened the probability of variation in both SBP and DBP. Replication of their technique was done using data used in this paper and the MAE was found as 9.8 mmHg for SBP and 9.3 mmHg for DBP. This experiment proves the necessity of a dataset with large variation in participants’ age and many records. The Framingham heart study [137], which followed blood pressure for more than 30 years, agreed that although SBP continuously increases as age goes from 30 to 84 and over, DBP slowly decreases after 50 and onward. In the study [138], out of all the participants, 17 were hypertensive and 12 were hypotensive. According to the Framingham study, the selection of age group from the participant will result in lower variation in DBP compared to SBP which explains the small value of MAE in DBP for [138]. Compared to those, the dataset used in this paper consisted of a much wider age range (20–100) as well as a higher number of patients [134]. In addition, in [138] the training and testing include measurement from the same 85 participants, which means the model has already “seen” those data before and might result in significant overfitting.

In addition, another significant criterion that was maintained while populating Table 4 is techniques or studies which used a large dataset with sufficient variations in data rather than the use of the selective and smaller dataset. In addition, using grading criteria proposed by BHS, the cumulative percentage and mean absolute error were measured with intervals as 5, 10, 15 mmHg. The minimum standard achieved by our proposed technique is grade B as shown in Table 4.

## 5. Conclusions and Discussion

The objective of this study was to propose and analyze the performance of a novel blood pressure measurement technique using an MSU-based optimized feature selection method combined with penalty-based regression. Researchers have been studying all relevant biomedical signals, such as ECG, PPG, PCG, BCG, etc., to extract necessary information and trying to come up with relations with change in blood pressure, but acceptable accuracy and consistency remain difficult to achieve. This paper proposed to improve accuracy and consistency by using a hybrid feature selection algorithm based on MSU so only relevant but nonredundant features from biomedical signals were used, this would make the input for the learning system optimized and reduce the chance of overfitting. The whole process initiates with biologically significant features from ECG and PPG signals which were extracted after necessary pre-processing steps. Using the advantage of both nonlinear and linear relations from multivariate symmetric uncertainty with the fast correlation and ReliefF algorithms, the optimized feature list was generated. Later, the finalized feature list was used for a penalty-based regularized regression to produce a general relation between the features and systolic/diastolic blood pressure. The regularized regression provides a balance between bias and variance to optimize the error or difference between the actual and predicted result.

In this study, the features were chosen based on their morphological features and their respective significance. To include statistical or spectral features there must be sufficient available information regarding how those features impact the change in blood pressure. Using the MIMIC II dataset, the regression model was trained and tested. The results showed improved performance in terms of RMSE and MAE compared to contemporary studies.

One significant limitation of this study is the assumption related to the arterial stiffness from the PPG wave pattern. Although this is not the ideal way, the assumptions were made to consider the effect the arterial stiffness without using additional sensors or invasive methods to measure arterial dimensions. Due to the assumptions, measurement accuracy was affected to some extent. The selection of features was done to compensate for that effect by choosing features specifically related to arterial stiffness. So, compared to other studies the effect of the assumptions was much lower in this study. It is a trade-off between assumptions and invasive sensors to get precise arterial information. Personal calibration with sufficient data should be an alternative solution for this trade-off.

Future work will use the developed measurement model and tune it using patient data for further calibration.

## Figures and Tables

**Table 1:** The initial feature list from the feature extraction phase.

Name of Feature	Detail of Feature
HR	Rate of heartbeat per minute
PPGMX & PPGMN	Maximum—Minimum amplitude of the peak value of the PPG wave
AC_amp and PPGIR	The magnitude of the AC part and ratio of Maximum—Minimum value of PPG
PTT	The time gap between corresponding ECG and PPG signal
S_T and DT	Systolic and diastolic time
Dias, D_S, D/S of_1_10	DT with one-tenth—maximum value, DT +ST with one-tenth—maximum value, Ratio of DT- ST with one-tenth of maximum value
Dias, D_S, D/S of_1_4	DT with one-fourth—maximum value, DT +ST with one-fourth—maximum value, Ratio of DT- ST with one-fourth of maximum value
Dias, D_S, D/S of_1_3	DT with one-third—maximum value, DT +ST with one-third—maximum value k, Ratio of DT- ST with one-third of maximum value
Dias, D_S, D/S of_1_2	DT with half of maximum value, DT +ST with half of maximum value, Ratio of DT- ST with half of maximum value
D_S__2_3 and 3_4	DT + ST with 2/3 and 3/4 of maximum value
ECG time interval and amplitude	QRS, P, T wave intervals and peak amplitudes

**Table 2:** The ranking based on Pearson’s correlation coefficient.

**SL**	**SBP**	**DBP**	**The linear correlation reduces →**
1	PPGIR	Heart rate	
2	Dias_1_10	PPGMN	
3	S_T	PPGIR	
4	D_S_1_10	PPGMX	
5	Dias_1_4	Dias_1_10	
6	AC_amp	D_S_1_10	
7	Dias_1_3	PTT	
8	D_S_1_4	D/S_1_10	
9	Dias_1_2	AC_amp	
10	PPGMN	Dias_1_4	
11	D_S_1_3	Dias_1_2	
12	D_S__1_2	D_S_1_4	

**Table 3:** Comparing the performance from different contemporary methods of BP measurement.

Method	MAE (mmHg)		Year
	SBP	DBP	
Using PTT [23]	6.85	6.35	2015
Peripheral PTT [136]	6.72	4.53	2016
Heart rate, pulse arrival time [135]	11.6	5.34	2017
PPT and IPG sensor [139]	8.55	5.07	2018
Feature from photoplethysmography wave [140]	9.42	6.87	2019
Using gradient boosting 1. and graph-theoretic algorithms [141]	8.09	5.49	2020
Regression using Pearson’s coefficients	9.50	10.10	2021
Proposed technique	4.27 (RMSE 5.28)	5.01 (RMSE 5.98)	2022

**Table 4:** Proposed method’s performance according to BHS.

BHS				
		CP—5 mmHg	CP—10 mmHg	CP—15 mmHg
BHS	Grade A	60%	85%	95%
	Grade B	50%	75%	90%
	Grade C	40%	65%	85%
Result from this study	SBP	65.29%	91.74%	99.87%
	DBP	58.37%	89.11%	99.63%

## Data Availability

Publicly archived datasets analyzed which can be found in the following website: https://archive.physionet.org/mimic2/ (accessed on 15 January 2021).
